# Human Leukocytes Kill *Brugia malayi* Microfilariae Independently of DNA-Based Extracellular Trap Release

**DOI:** 10.1371/journal.pntd.0005279

**Published:** 2017-01-03

**Authors:** Ciaran J. McCoy, Barbara J. Reaves, Steeve Giguère, Ruby Coates, Balázs Rada, Adrian J. Wolstenholme

**Affiliations:** 1 Department of Infectious Diseases, College of Veterinary Medicine, University of Georgia, Athens, GA, United States of America; 2 Center for Tropical and Emerging Global Diseases, University of Georgia, Athens, GA, United States of America; 3 Department of Large Animal Medicine, College of Veterinary Medicine, University of Georgia, Athens, GA, United States of America; 4 Department of Biology & Biochemistry, University of Bath, Bath, United Kingdom; Queen's University Belfast, IRELAND

## Abstract

**Background:**

*Wuchereria bancrofti*, *Brugia malayi* and *Brugia timori* infect over 100 million people worldwide and are the causative agents of lymphatic filariasis. Some parasite carriers are amicrofilaremic whilst others facilitate mosquito-based disease transmission through blood-circulating microfilariae (Mf). Recent findings, obtained largely from animal model systems, suggest that polymorphonuclear leukocytes (PMNs) contribute to parasitic nematode-directed type 2 immune responses. When exposed to certain pathogens PMNs release extracellular traps (NETs) in the form of chromatin loaded with various antimicrobial molecules and proteases.

**Principal findings:**

*In vitro*, PMNs expel large amounts of NETs that capture but do not kill *B*. *malayi* Mf. NET morphology was confirmed by fluorescence imaging of worm-NET aggregates labelled with DAPI and antibodies to human neutrophil elastase, myeloperoxidase and citrullinated histone H4. A fluorescent, extracellular DNA release assay was used to quantify and observe Mf induced NETosis over time. Blinded video analyses of PMN-to-worm attachment and worm survival during Mf-leukocyte co-culture demonstrated that DNase treatment eliminates PMN attachment in the absence of serum, autologous serum bolsters both PMN attachment and PMN plus peripheral blood mononuclear cell (PBMC) mediated Mf killing, and serum heat inactivation inhibits both PMN attachment and Mf killing. Despite the effects of heat inactivation, the complement inhibitor compstatin did not impede Mf killing and had little effect on PMN attachment. Both human PMNs and monocytes, but not lymphocytes, are able to kill *B*. *malayi* Mf *in vitro* and NETosis does not significantly contribute to this killing. Leukocytes derived from presumably parasite-naïve U.S. resident donors vary in their ability to kill Mf *in vitro*, which may reflect the pathological heterogeneity associated with filarial parasitic infections.

**Conclusions/Significance:**

Human innate immune cells are able to recognize, attach to and kill *B*. *malayi* microfilariae in an *in vitro* system. This suggests that, *in vivo*, the parasites can evade this ability, or that only some human hosts support an infection with circulating Mf.

## Author Summary

Lymphatic filariasis is a disfiguring and debilitating neglected tropical disease caused by filarial parasitic nematodes including *Brugia malayi*. The immune mechanisms that drive host tolerance and host resistance to blood circulating microfilariae are not yet fully understood. In this study, we investigate the interaction between *B*. *malayi* microfilariae and human peripheral blood leukocytes. Polymorphonuclear leukocytes are the most abundant leukocyte population present within the human circulatory system and can release DNA-based extracellular traps (NETs) that capture and kill specific pathogens. We show that human neutrophils release NETs in response to *B*. *malayi* parasites. These NETs promote leukocyte-to-worm attachment but do not kill the microfilariae. Despite this, we highlight that neutrophils and monocytes can kill these parasites *in vitro*. Interestingly, the population of healthy human blood donors appeared to split into two distinct phenotypes: “Mf killers” and “non-killers”. Surprisingly, our findings suggest that activation of the complement system does not significantly contribute to leukocyte mediated parasite killing. Our study provides new insights into host-microfilariae interactions and the role that the human innate immune system plays in filarial parasite defense and the regulation of microfilaremia, a key determinant of disease transmission.

## Introduction

Throughout history, parasitic nematode infections have had a major impact on human development, especially of the poorest and most disadvantaged populations. Human diseases associated with these infections include lymphatic filariasis (LF) and onchocerciasis. A hallmark of these infections is that they are very long-lasting, with the production of very large numbers of microfilariae (Mf) that are able to survive within the host. In order to do this, parasitic nematodes have evolved the ability to modulate and suppress the host immune response via the secretion of a cocktail of proteins, micro RNAs and small molecules [1–4]. The current strategy for the elimination of LF and onchocerciasis as public health problems centers on the prevention of transmission by eliminating the Mf from infected hosts, thus preventing any new infections of the insect vectors and hence, more human hosts [5–7]. At present, this is achieved by mass administration of the effective anthelmintic drugs, albendazole, ivermectin and diethylcarbamizine (DEC). Studies on DEC have indicated that the drug interacts with the host immune system in order to be effective [8–13] and some experiments with animal parasites have suggested that ivermectin has an impact on the ability of polymorphonuclear leukocytes (PMNs) and monocytes to attach to and kill Mf [14, 15]. These results imply that host granulocytes and monocytes have the ability to recognize Mf and possibly to kill them.

The innate immune response to parasitic nematodes involves many different cell populations, which include granulocytes such as eosinophils, mast cells and PMNs, as well as monocytes. PMNs are critical for controlling a large variety of pathogens including nematodes. They have previously been implicated in the killing of nematode larvae, including *Onchocerca volvulus* Mf and L3 [16, 17], and have been reported to be a key component of the host innate immune response to nematode infections [18]. For example, increased numbers of PMNs in the skin and blood of infected mice reduced the success of invading L3 of the filarial nematode, *Litomosoides sigmodontis* [19]. A characteristic feature of PMN responses is the production of DNA-containing neutrophil extracellular traps (NETs) [20]. These structures are formed by a unique type of cell death, NETosis, and are characterized by large, extracellular concentrations of expelled cytosolic, granular and nuclear material including DNA, histones, neutrophil elastase and myeloperoxidase [21]. NETosis is frequently, but not always, mediated by NADPH oxidase [21–22]. NET formation is induced by parasitic nematodes but whether these are required for nematode killing is uncertain and may depend on the parasite under study. Despite being trapped by NETs *in vitro*, the L3 larvae of both *Strongyloides stercoralis* and *Haemonchus contortus* were not killed by NETs alone [23–24] although treatment with DNase to destroy NETs did reduce PMN plus macrophage mediated killing of *S*. *stercoralis* L3 [23]. In several studies PMNs have been shown to co-operate with monocytes or macrophages in immunity against parasites, including helminths [18, 24–27].

We have previously shown that PMNs and peripheral blood mononuclear cells (PBMCs) from uninfected dogs attach to *Dirofilaria immitis* Mf *in vitro* and that this attachment was increased by the addition of ivermectin [14]. We have extended these studies to the human parasite *Brugia malayi* and investigated the ability of leukocytes purified from presumably parasite-naïve North American human donors to recognize and kill *B*. *malayi* Mf isolated from the peritoneal cavity of infected Mongolian gerbils, *Meriones unguiculatus*. *B*. *malayi* is the causative agent of a minority (roughly 10%) of cases of LF, however it is the only filarial nematode of humans that can be maintained in a convenient laboratory animal host. Our results provide evidence that PMNs and monocytes of many, but not all, human donors were able to both adhere to and kill *B*. *malayi* Mf.

## Results

### Human PMNs release NETs that entangle *B*. *malayi* Mf *in vitro*

**Fig 1:**
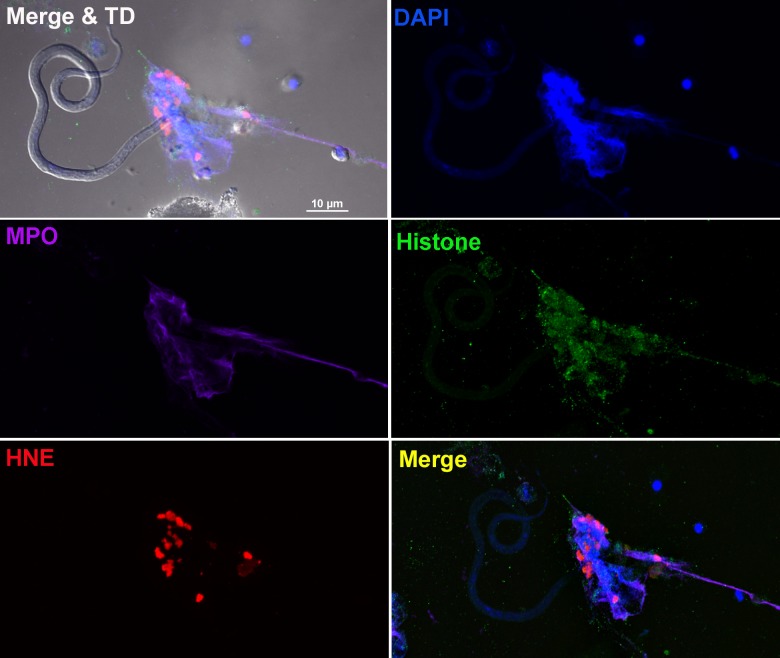
*B*. *malayi* Mf entangled within neutrophil extracellular traps. Fluorescence imaging of a worm-NET aggregate labelled with DAPI (blue) and antibodies to myeloperoxidase (MPO; purple), citrullinated histone H4 (histone; green) and human neutrophil elastase (HNE; red). The co-localization of markers with Mf is shown in the merged image with transmitted light (top left panel) and the overlap of MPO, histone and HNE with DNA shown in the merged image (bottom right panel). Live Mf were incubated in the presence of human neutrophils for 18 hours at 37°C and 5% CO_2_. Scale bar = 10μm.

Although NET formation was initially characterized in response to bacteria and protozoa [20, 27], it has also been reported for larger multi-cellular pathogens including fungi and three species of parasitic nematodes [19, 24, 25]. Our initial experiments co-culturing *B*. *malayi* Mf with human neutrophils in the presence of the membrane-impermeable DNA-binding dye SYTOX Orange resulted in Mf becoming tethered in a manner consistent with entanglement in NETs (S1 Video). These observations prompted us to confirm that the structures generated possessed typical NET characteristics. Live Mf were co-cultured with human PMNs and the generation of NETs observed by confocal microscopy after staining with DAPI and antibodies to characteristic NET-associated proteins. Worm-NET aggregates stained positive for citrullinated histone H4 and the granular proteins, neutrophil elastase and myeloperoxidase (Fig 1). All the NET-associated proteins examined co-localized with extracellular DNA, confirming typical NET morphology.

### Mf induce the release of PMN-derived extracellular DNA

**Fig 2:**
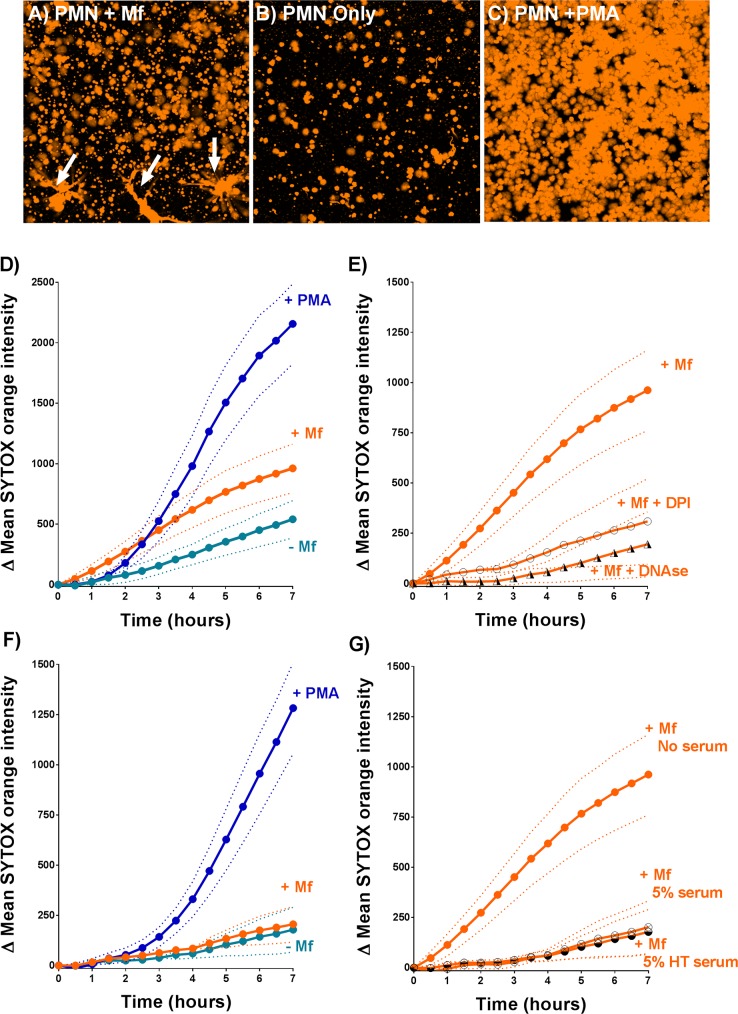
Extracellular DNA release from human PMNs. A-C: Images of SYTOX Orange-labelled extracellular DNA present within Mf-treated (A), zero Mf (negative control) (B) and PMA-treated (positive control) (C) wells. The images were taken 7 hours post-assay set up. These wells did not contain serum. D-G: Changes in mean SYTOX Orange intensity were used to monitor the release of extracellular DNA from PMNs incubated for 7 hours *in vitro*. In each panel, the dotted lines indicate the Standard Error of the mean fluorescence intensity. D: 25nM phorbol myristate acetate (PMA)- and Mf-induced DNA release in the absence of serum was measured as described in Materials and Methods (n = 7). E: 10μM diphenyleneiodonium (DPI) and 30μg/ml DNAse I inhibited Mf-induced DNA release in the absence of serum (n≥4). F: Extracellular DNA release in Mf- and PMA-treated wells that contained 5% autologous serum (n = 6). G: 5% autologous serum and 5% autologous heat treated serum (HTS) inhibited Mf induced DNA release (n≥6).

To further characterize Mf-induced NETosis, we developed a confocal microscopy-based extracellular DNA release assay. Live cell fluorescence imaging of PMN and Mf interactions in the presence of SYTOX Orange allowed us to observe extracellular DNA and NET formation over time (Fig 2A–2C and S2 Video). Mean SYTOX Orange intensity values derived from these images were used to quantify total extracellular DNA release (Fig 2D–2H) in wells where PMNs were stimulated with either Mf or 25nM phorbol myristate acetate (PMA) as a positive control [28]. These data demonstrate that in the absence of serum, *B*. *malayi* Mf significantly increased the release of extracellular DNA when compared to the zero Mf controls (Fig 2D; P = 0.041). Both DNase I and the NADPH oxidase inhibitor diphenyleneiodonium (DPI) significantly reduced the mean SYTOX Orange intensities derived from Mf treated wells (Fig 2E; P<0.001 for both treatments), presumably via the enzymatic breakdown of NET structure and the inhibition of NETosis respectively. Interestingly, in the presence of 5% autologous serum, we did not detect any significant increase in extracellular DNA release within Mf treated wells compared to the zero Mf controls (Fig 2F). This may be due to the inhibitory effects of the autologous sera which impeded DNA release in Mf (P<0.001) containing wells (Fig 2G). The autologous sera also reduced the extracellular DNA release induced by PMA (S1 Fig). Heat treatment of the autologous sera had no significant effect (Fig 2G), suggesting that the complement system was not responsible for the inhibition of extracellular DNA release.

### Autologous serum and extracellular DNA promote PMN to worm attachment

**Fig 3:**
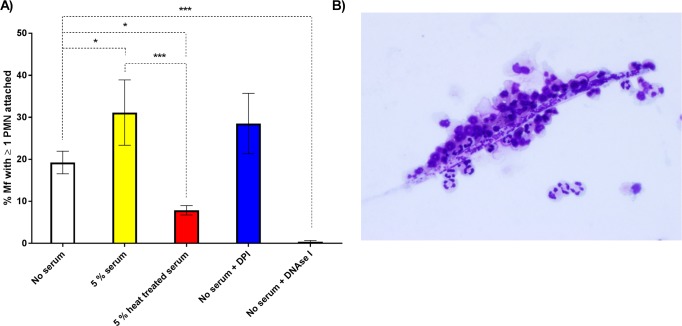
The attachment of PMNs to *B*. *malayi* Mf. A: The percentage of Mf that had at least one PMN adhered to their surface or indirectly fastened by extracellular DNA at 24 hours post-experimental set up (n≥6). White, yellow, red, blue, and black bars represent no serum, 5% autologous serum, 5% autologous heat-treated (55°C, 30 mins) serum, 10μM diphenyleneiodonium (DPI) and 30μg/ml DNase I treated wells respectively. Error bars represent standard error of the mean; *P<0.05, **P<0.01, ***P<0.001. B: Image of PMNs adhered to a Mf. Image taken after 24 hours of incubation in 5% autologous serum at 37°C and 5% CO_2_. The preparation was stained with modified Wrights’ stain.

We have previously shown that canine neutrophils isolated from uninfected dogs can attach to *D*. *immitis* Mf *in vitro* [14]. Therefore, we examined whether a similar phenomenon was observed when we incubated PMNs from uninfected humans with Mf of the human parasite, *B*. *malayi*. Video analysis of 96-well plate-based co-cultures allowed us to confirm PMN to Mf attachment *in vitro* and to count the number of individual Mf with at least one cell attached. After 24 hours, the addition of 5% autologous serum increased the number of worms with attached PMNs from 19.3% to 31.1% (P = 0.035). DNase I treatment virtually abolished PMN attachment in the absence of serum (0.4 ± 0.3% of Mf had ≥1 PMN attached at 24 hours post experimental set up (p<0.001),), suggesting that NETs were required for PMNs to attach to the worms (Fig 3A). In contrast, DPI had no significant impact on PMN attachment under these conditions (Fig 3A), suggesting that attachment took place via an NADPH oxidase-independent mechanism. Heat treatment (55°C for 30 min) of the sera inhibited attachment, reducing it to levels less than in the zero serum controls, with only 7.9% of the Mf having at least one cell attached (P = 0.015) (Fig 3A). The effect of heat treatment suggested that heat-labile components of the autologous serum promoted PMN to worm attachment. The number of PMNs attached to individual Mf varied, and we often observed Mf that had large numbers of PMNs adhered to their surface (Fig 3B); however, only a single cell was required to be attached in these assays for the Mf to be scored.

### Human leukocytes can kill *B*. *malayi* Mf *in vitro*

**Fig 4:**
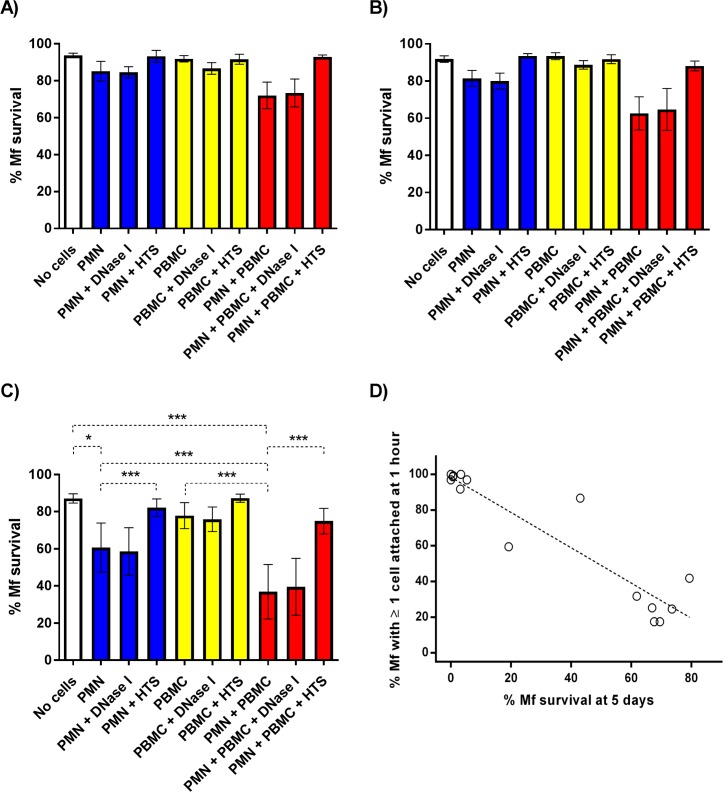
Mf survival in the presence of PMNs and PBMCs. The percentage of Mf that survived to days 1 (A), 2 (B) and 5 (C) post-experimental set up (n = 5). White, blue, yellow, and red bars represent zero cell, 1500 PMN/Mf, 1500 PBMC/Mf and PMN plus PBMC treated wells respectively. All wells contained either 25% autologous serum or 25% heat treated serum (HTS). DNase I denotes the addition of 30μg/ml DNase I. Error bars represent standard error of the mean; *P<0.05, **P<0.01, ***P<0.001. D. Scatter plot comparing leukocyte attachment and Mf survival in the presence of PMNs and PBMCs. The percentage of Mf that had at least one PMN/PBMC adhered to their surface or indirectly fastened by extracellular DNA at 1 hour post-experimental set up plotted against the percentage of Mf that survived to day 5 (n = 15). Each point represents an individual biological replicate derived from distinct human blood donors. All wells contained 25% autologous serum, ~100 Mf, ~150,000 PMNs and ~150,000 PBMCs.

The previous experiments clearly showed that human PMNs can recognize and attach to *B*. *malayi* Mf. Bonne-Année and colleagues reported that PMNs and peripheral blood mononuclear cells (PBMCs) collaborate to kill *S*. *stercoralis* larvae when incubated in 25% human serum for 48 hours [29], so we tested whether or not the formation of NETS and cell attachment we observed also resulted in Mf killing. Worm survival was monitored over 5 days in culture in the presence and absence of peripheral blood leukocytes (PMNs and PBMCs) isolated from uninfected people. Approximately 85% of Mf survived for 5 days in the absence of human leukocytes (Fig 4C). Exposure to PBMCs alone did not significantly alter Mf survival *in vitro* (Fig 4), but when Mf were maintained in the presence of either PMNs alone or both PMNs and PBMCs, worm survival was significantly inhibited compared to the zero cell controls (Fig 4). Interestingly, when Mf were incubated with both PMNs and PBMCs, significantly fewer worms survived to day 5 (36.9 ± 14.7%, see Fig 4C; P<0.001) when compared to the Mf exposed to PMNs alone (60.6 ± 14.7%, see Fig 4C; P<0.001). DNase I treatment of the cultures had no significant effect on Mf survival in the presence of PMNs, PBMCs or both, indicating that NET formation was not required for Mf killing, whereas heat treatment of the serum effectively blocked all leukocyte-mediated killing (Fig 4A–4C).

Over the course of these experiments we noticed that the levels of both cell to worm attachment and leukocyte mediated Mf killing varied greatly between individual experiments. In an attempt to better understand this variation we re-analyzed the videos of co-culture wells that contained both PMNs and PBMCs to score leukocyte to worm attachment and compare this to Mf survival. These data highlight an obvious negative correlation between the levels of leukocyte attachment at one hour post experimental set up and the percentage of worms that survived to day 5 (Fig 4D; Spearman's rho = -0.85, P<0.0001). Each experiment was carried out using cells from a single donor and this donor was different for each experiment. The isolated cells appeared to split largely into two distinct phenotypes: “Mf killers” who displayed both rapid leukocyte attachment (>90% Mf with ≥1 leukocyte attached at 1 hour) and near complete Mf killing (<10% of the Mf survived to day 5) and “non-killers” who displayed relatively low levels of leukocyte attachment (<42% Mf with ≥1 leukocyte attached at 1 hour) and little to no leukocyte mediated Mf killing compared to zero cell controls (60–80% Mf survival at 5 days post set up), though there were 2 donors whose cells had an intermediate phenotype. This explains the relatively high variation (reflected in the error bars) in attachment and killing seen between experiments. This analysis also reveals that when killing occurs the leukocytes recognize and attach to the Mf very rapidly and that if this attachment does not take place within one hour, the parasites survive quite well in these culture conditions.

### The complement system does not promote PMN attachment or leukocyte-mediated Mf killing

**Fig 5:**
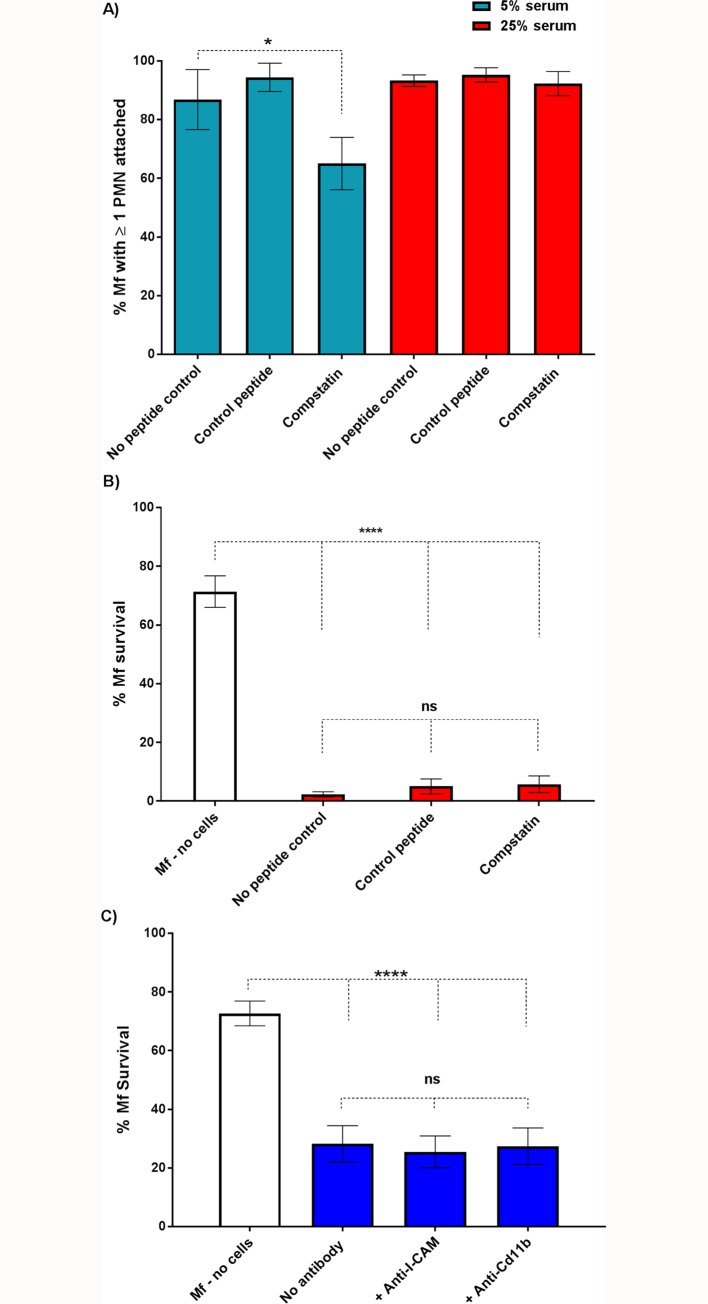
Compstatin-mediated inhibition of complement activation has little effect on Mf survival. A: The percentage of Mf that had at least one PMN adhered to their surface or indirectly fastened by extracellular DNA at 24 hours post-experimental set up (n = 3). Orange and red bars represent 5% autologous serum and 25% autologous serum treated wells respectively. The autologous serum was either untreated (no peptide control), treated with 100μM inactive compstatin analogue (control peptide) or treated with 100μM active compstatin. Error bars represent standard error of the mean. B: The percentage of Mf that survived 5 days in the presence of both PMNs and PBMCs after treatment of serum with control peptide or compstatin (n = 3). C: PMNs were incubated with blocking antibodies for 2 hours prior to incubation with Mf for 5 days. PMN-mediated killing occurred to the same extent in control (no antibody) wells and those treated with anti-ICAM-1 and anti-Cd11b (p = <0.0001). No significant differences were observed between PMN incubated with Mf with or without the anti-ICAM-1 or anti-Cd11b (n = 3).

Serum heat inactivation has been shown to prevent the killing of *S*. *stercoralis* L3 larvae mediated by human PMNs and PBMCs [29]. These observations led to the conclusion that complement was required for larval killing. Given the effects of serum heat treatment on PMN attachment (Fig 3) and subsequent Mf survival (Fig 4), we directly investigated the possibility that the complement system is involved in PMN to worm attachment and leukocyte-mediated Mf killing. We repeated our PMN attachment and Mf survival assays whilst blocking complement activation via the complement specific inhibitor, compstatin [30]. In these experiments we only analyzed data obtained from those experiments where substantial Mf killing was observed, since this is the phenomenon we were seeking to study. Pre-treatment of 25% serum with 100μM compstatin did not significantly affect either attachment (Fig 5A, red panels) or killing (Fig 5B). These data suggest that the complement system does not significantly contribute to PMN plus PBMC mediated Mf killing *in vitro*. However, since the attachment experiments were conducted in 5% serum we also tested the effect of compstatin on attachment under these conditions. Although there was a small reduction between the control peptide (~90% attachment) and the compstatin treated samples (~65% attachment) (Fig 5A, light blue panels, P = 0.045), there was no significant difference in PMN to worm attachment between the no peptide control and compstatin treated wells. To further investigate the role of complement we also used blocking antibodies to two complement- and adhesion-associated proteins, Cd11b (complement receptor 3, CR3) and ICAM-1. Neither blocking antibody had an effect on Mf survival (Fig 5C).Complement therefore plays only a minor role, if any, in attachment and killing of Mf, though a heat-labile component of normal human serum is required.

### Monocyte-mediated Mf killing

**Fig 6:**
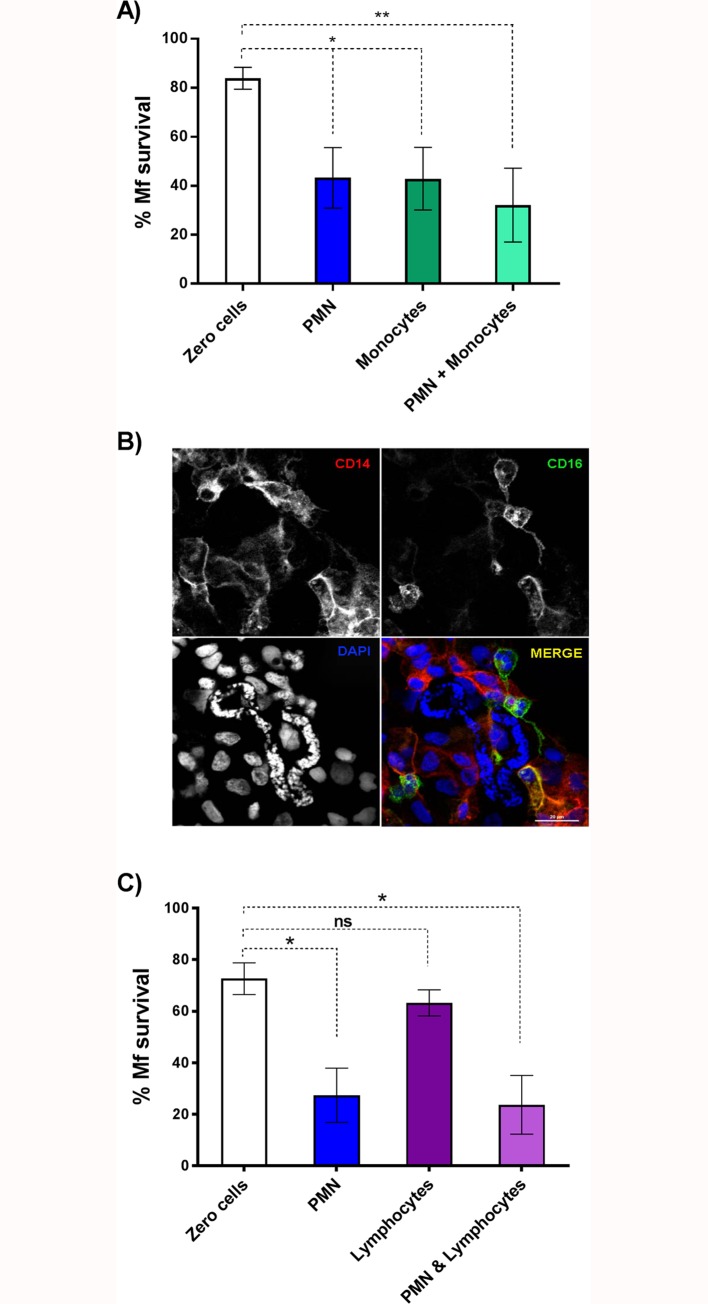
Mf survival in the presence of human PMNs and monocytes. A: The percentage of Mf that survived to day 5 (n≥7). White, blue, green, and light green bars represent no cell, 1500 PMN/Mf, 1500 monocytes/Mf and 1500 PMN/Mf plus 1500 monocytes/Mf treated wells respectively. Error bars represent standard error of the mean; *P<0.05, **P<0.01, ***P<0.001. B: CD14^+^ and CD16^+^ cells attach to *B*. *malayi* Mf in culture. Mf were co-incubated with both PMNs and monocytes for 5 days and processed for immunofluorescence microscopy as described in Materials and Methods. Monocytes were isolated using negative immunoselection for CD16 and therefore are CD14 positive and CD16 negative. These are detected with anti-CD14-Alexa fluor 598 (red). PMNs express both CD14 and CD16 so are identified by both anti-CD14 (red) and CD16 (green) labelling. DAPI staining of Mf nuclei is indicated by a yellow arrow, highlighting the position of the Mf. Bar = 20 μM. C: The percentage of mf that survived to day 5 in the absence of immune cells (white bar), or in the presence of 1500 PMN (blue bar), 1500 lymphocytes (purple bar) or 1500 PMN plus 1500 lymphocytes (light purple bar). Error bars represent standard error of the mean; *P<0.05 (n = 3).

Addition of PBMCs to PMNs increased the amount of Mf killing, however, PBMCs alone were not sufficient to affect survival. Monocytes and neutrophils are both crucial in immune responses to infection [31] and interactions important for helminth clearance have recently been described [18]. Monocytes make up ~10% of the PBMC preparation isolated for our survival experiments (see Materials and Methods) so we hypothesized that monocytes may represent the microfilaricidal cells present within the PBMC population. To test this, we repeated our Mf survival assays but replaced the PBMC population with 1500 monocytes/Mf. In these experiments, incubation with either PMNs or monocytes reduced Mf survival after 5 days to about 40%; incubation with both cell types did not significantly reduce survival any further (Fig 6A) although attachment of both PMN and monocytes could be detected in co-cultures at 120 hours post incubation (Fig 6B). These data highlight that both PMNs and human monocytes are independently capable of killing *B*. *malayi* Mf *in vitro* (P = 0.005). Immunofluorescence staining showed that both CD14^+^/CD16^-^ monocytes and CD14^+^/16^+^ PMNs were attached to the Mf at 5 days post-infection by which time significant levels of killing had occurred (Fig 6B). In contrast, purified lymphocytes were unable to kill Mf, nor did their addition to PMN reduce survival any further (Fig 6C).

Taken together, our data show that PMNs and monocytes, but not lymphocytes, isolated from North American human donors, who have presumably never been exposed to *B*. *malayi*, can recognize and kill Mf *in vitro*. There is some variation in the extent of parasite killing between experiments, which may represent differences between the cells isolated from individual human donors, or between batches of Mf. Killing is preceded by rapid attachment (<1 hr) of the leukocytes, but is rather slow, taking up to 5 days.

## Discussion

Filarial nematodes, including Mf, survive for months and years in their hosts without provoking an effective immune response. Nonetheless, in this paper we confirm that leukocytes taken from uninfected people can recognize, attach to and kill the Mf stage *in vitro* [15–17]. As a starting point we wished to determine if the results we previously obtained using the animal parasite, *D*. *immitis*, and canine leukocytes [14], could be reproduced *in vitro* using human cells and a human filarial parasite. In particular, we wanted to extend these observations and determine if human PMN-derived DNA-based extracellular traps (NETs) [32] could ensnare and kill the Mf of *B*. *malayi*. NETosis remains poorly characterized with respect to immunity to parasitic nematodes. Recent studies have confirmed that NETs are released from human, bovine and mouse PMNs exposed to the L3 larval stages of *S*. *stercoralis*, *H*. *contortus* and *L*. *sigmodontis* respectively [19, 24, 25]. Both *H*. *contortus* and *S*. *stercoralis* L3 larvae were trapped but not killed by NETs alone [24, 25], though DNase I-mediated extracellular trap destruction prevented human PMN-, macrophage- and autologous serum-mediated killing of *S*. *stercoralis* larvae. Extracellular traps may therefore contribute to broader killing mechanisms that require multiple immune components. Mouse neutrophils release NETs when exposed to larvae of the human parasite, *S*. *stercoralis* [24], though DNase I treatment did not block killing of the larvae by mouse leukocytes *in vitro* [24]. Our confocal microscopy-based assays confirmed the presence of classical NET markers when human PMNs were incubated with *B*. *malayi* Mf, demonstrating that Mf can induce NET release *in vitro* (Fig 1 and Fig 2A–2D), and that these structures contain all of the components reported from other systems [20, 21]. DNase I treatment effectively destroyed NET structure (Fig 2E) and blocked PMN to Mf attachment in the absence of serum (Fig 3A), as predicted, but did not inhibit human leukocyte mediated Mf killing (Fig 4C). DNA-containing NET formation is therefore not essential for human leukocytes to kill *B*. *malayi* Mf *in vitro*. This suggests that the importance of NETosis to nematode parasite killing varies with both host and parasite species. It is also possible that *in vivo* NETs do contribute to Mf killing but that additional components were missing from our *in vitro* survival assay. An increase in NET-like structures was correlated with reduced *S*. *stercoralis* L3 survival within cell impermeable diffusion chambers implanted into the mouse model [24]. DPI significantly inhibited *B*. *malayi* Mf-induced DNA release in the absence of autologous serum (Fig 2E), mirroring the results associated with *H*. *contortus* [25] and indicating a role for NADPH oxidases in parasitic nematode driven NETosis. Despite this, DPI appeared to have no significant impact on PMN to Mf attachment (Fig 3A) and obvious NET aggregates could be seen within DPI treated wells. We were not able to examine the effect of DPI on Mf survival due to the negative effects of long term DPI exposure on worm health (the worm appeared sluggish but not dead at day 5); these effects are presumably due to DPI inhibiting the nematodes’ NADPH oxidase.

Human PMNs can attach to and kill *B*. *malayi* Mf *in vitro*, and Mf survival is further reduced if PBMCs are added to the cultures (Fig 4). The PBMC population failed to kill Mf in the absence of other cell types, yet significantly increased the level of parasite killing when co-cultured with PMNs (Fig 4), suggesting some cross-talk between PMNs and a component of the PBMC fraction, presumably monocytes since these cells are also able to kill Mf (Fig 6). We noted a large amount of variation in both leukocyte attachment and Mf survival between individual experiments, and the two measurements–attachment at 1 hour and survival at 5 days–are clearly negatively correlated (Fig 4D). This could arise from differences between the cells and/or serum isolated from individual human donors, or between different batches of Mf. It is impossible to distinguish between the two using our current protocols as we do not know the identity of the human donors, and so cannot examine HLA genotypes for example, and since cells and sera were used immediately after isolation, we could not test them on different batches of Mf. In endemic regions the majority of infected individuals are tolerant of high parasite loads and microfilaremia. In contrast, individuals with pathological manifestations (e.g. lymphedema and hydrocele) show stronger immune reactions [33]. The genetic factors that regulate susceptibility to parasitic infections and the pathological heterogeneity associated with filarial nematode infection are not entirely understood [34], but it is possible that these are reflected in the ability of the innate immune system to rapidly recognize and kill Mf, as shown here. In contrast, the nematodes used in this study are an inbred population that would not be expected to exhibit much genetic variation, but non-genetic factors may account for the differences observed between experiments. For example, differences in the amounts of immunomodulatory ES products present in the various batches of Mf may explain the variation in rapid attachment and subsequent killing that we observed.

PMN attachment and Mf killing were both promoted by the addition of autologous serum, and heat treatment of the serum inhibited both PMN attachment and PMN plus PBMC-mediated Mf killing (Fig 3 and Fig 4). This was not due to complement as the complement specific inhibitor compstatin failed to have any biologically significant effects on either PMN attachment or leukocyte mediated Mf killing (Fig 5). Compstatin binds to C3 to prevent C3 cleavage and competitively inhibit all three complement activation pathways [30]. These data suggest that complement does not contribute much, if at all, to PMN attachment or Mf killing, but that another unidentified heat-labile component of human serum is involved. Antibodies against CD11b and ICAM-1, two molecules previously implicated in the interactions between Mf and the immune system [18, 33], also failed to inhibit killing, suggesting that they are not required for this process to take place (Fig 5C).

In the presence of autologous serum both PMNs and monocytes are capable of killing Mf alone (Fig 4 and Fig 6). Bonne-Année and colleagues have shown that human PMNs can collaborate with either PBMCs or macrophages to kill *S*. *stercoralis* larvae [24, 29], however, despite using relatively high numbers of leukocytes, they did not observe reduced L3 larvae survival on exposure to individual leukocyte populations [24, 29]. This could reflect differences in the nematode species or life-stages employed, particularly worm size which varies considerably between life-stages and perhaps influences the number of leukocytes required to kill the parasite. Perhaps surprisingly, we observed no increased killing when PMNs and monocytes were incubated together with the Mf. Neutrophils and monocytes are well known to communicate with each other and it is has been reported that neutrophils induce an anti-nematode immunity in monocytic cells [18, 35], however this was not reflected in our *in vitro* experimental system.

These data describe the first example of Mf induced NETosis and contribute significantly to the growing body of evidence that suggest an important role for neutrophils in regulating parasitic nematode infections [18, 36]. We show that human peripheral blood innate immune cell populations can recognize, trap and kill the blood circulating life-cycle stage of the human filarial parasitic nematode, *B malayi*. Variation in worm killing and leukocyte attachment between human blood donors suggest that the innate immune system could significantly contribute to the regulation of host tolerance and susceptibility to infection and more specifically the regulation of microfilaremia which is a key determinant of the transmission of lymphatic filariasis.

## Materials and Methods

### Ethics statement

All experiments and informed consent procedures were approved by the Institutional Review Boards of the University of Georgia (permit number 2012–10769), and the studies were conducted in accordance with the ethical guidelines of Declaration of Helsinki. Human subjects recruited under the guidelines of IRB-approved protocols provided written informed consent for participation in the studies described below.

### Microfilariae preparation

Live *B*. *malayi* Mf isolated from the peritoneal cavity of infected Mongolian gerbils were provided by the Filarial Research Reagent Resource Center (FR3: Athens, GA, USA). Mf were washed three times in phosphate buffered saline (PBS; centrifuged at 1500 x g for 8 min) and re-suspended in RPMI-1640 (Gibco, Life Technologies, Grand Island, NY, USA). Note that all RPMI-1640 used in this study was supplemented with 100 U/ml penicillin-streptomycin (Life Technologies, Grand Island, NY, USA) and 0.1 mg/ml gentamicin (Sigma, St. Louis, MO, USA). Re-suspended Mf samples were then filtered through a 5μm Isopore membrane (Merck Millipore Ltd., Carrigtwohill, Cork, Ireland) to capture the Mf and exclude contaminating small particles. Membranes were socked in RPMI-1640 at 37°C and 5% CO_2_ for 20–30 min to facilitate the migration of viable Mf from the membrane. Viable Mf were incubated overnight in RPMI-1640 at 37°C and 5% CO_2_. Mf samples were washed for a second time by 5μm Isopore membrane filtration just before use.

### Isolation of human neutrophils, PBMCs, monocytes and lymphocytes

Leukocytes were isolated from freshly donated peripheral blood drawn from healthy U.S. residents at the Health Center of the University of Georgia. 40ml of blood was anticoagulated by heparin. PMNs were isolated using the EasySep Direct Human Neutrophil Isolation Kit (Stemcell Technologies, Vancouver, BC, Canada) according to manufacturer’s instructions. PBMCs were isolated using SepMate-50 Tubes (Stemcell Technologies, Vancouver, BC, Canada) according to manufacturer’s instructions. The optional extended wash step (120 x g for 10 min) of the SepMate protocol was included to remove contaminating platelets. Monocytes were isolated from the PBMC samples using the EasySep Human Monocyte Enrichment Kit (Stemcell Technologies, Vancouver, BC, Canada) according to manufacturer’s instructions. Isolated PMNs, PBMCs and monocytes were washed in PBS (centrifuged at 300 x g for 5 min), re-suspended in a 1:1 mixture of RPMI-1640 and autologous serum, stored at room temperature and used within 6 hours post-isolation as previously described [37]. The concentration of cells per population was estimated in a sample of cells stained with 0.4% trypan blue (Gibco, Life Technologies, Grand Island, NY, USA) using a hemocytometer. All populations used were estimated at 95% or greater viability. To estimate the purity of isolated cell populations, cell slides were prepared using the Cytospin 3 CellPreparation System (Shandon Scientific Limited, Astmoor, Runcorn, Cheshire, England) and stained with Modified Wright’s stain (Hema 3 Stat pack, Fisher Scientific, Kalamazoo, MI). The average purity of the PMN population was ~97% and the monocyte population ~92%. The PBMC population contained predominantly lymphocytes but included ~10% monocytes and ~10% PMNs. Leukocytes were washed once in Hanks-balanced salt solution (HBSS; Gibco, Life Technologies, Grand Island, NY, USA; centrifuged at 300 x g for 5 min) and re-suspended in RPMI-1640 before use. Lymphocytes were isolated using the EasySep Direct Human Total Lymphocyte Isolation Kit (Stemcell Technologies, Vancouver, BC, Canada) according to manufacturer’s instructions. Isolated lymphocytes were washed in PBS (centrifuged at 300 x g for 5 min) and re-suspended in a 1:1 mixture of RPMI-1640 and autologous serum.

### Autologous human sera

10 ml of autologous blood was collected as described above but allowed to clot in the absence of heparin (incubated for ~2 hours at room temperature) [38]. Briefly, the liquid fraction of the blood sample was aspirated and centrifuged at 10,000 x g for 5 min. The supernatant was aspirated and filter sterilized. Where necessary the serum was heat treated (55°C for 30 min) and/or diluted in RPMI-1640 to the desired concentration before use.

### Immunostaining and confocal microscopy of NETs and Mf-associated leukocytes

For detection of NET-associated proteins, isolated PMNs, 1.5 x 10^5^ cells in 100 μl RPMI-1640, were seeded onto 12 mm #1 round coverslips in 24-well flat bottom dishes (Costar, Corning, NY, USA) and left to adhere for 1 hour at 37°C and 5% CO_2_ prior to adding 100 *B*. *malayi* Mf in 100 μl RPMI-1640. The coverslips were incubated a further 18 hours at 37°C and 5% CO_2_ prior to immunostaining. Cells and Mf were fixed by adding 200 μl of 4% paraformaldehyde to the well and incubated for 20 min at room temperature. Supernatants were carefully removed and coverslips washed twice with PBS prior to blocking with PBS plus 5% FBS and 1% BSA for 20 min at room temperature. Primary antibodies were diluted in blocking solution as follows: (1) rabbit anti-histone H4 (citrulline R3) antibody 1:500 (ab81797; Abcam, Cambridge, MA, USA), (2) mouse anti-human myeloperoxidase 1:200 (clone MPO455-8E6; eBioscience, San Diego, CA, USA) and (3) goat anti-human neutrophil elastase 1:200 (clone C-17, sc-9520; Santa Cruz Biotechnology, Dallas, TX, USA). After 1 hour incubation at room temperature, coverslips were washed three times with PBS and incubated with a 1:200 dilution of donkey anti-rabbit IgG-Alexa488, donkey anti-goat IgG-Alexa555 and donkey anti-mouse IgG-Alexa647 (Life Technologies, Thermo Fisher Scientific) in blocking buffer containing 0.1μg/ml DAPI for 30 min at room temperature. Coverslips were washed, mounted in Mowiol (Calbiochem/EMD Biosciences, La Jolla, CA) and imaged using a Nikon A1R confocal microscope (Nikon Instruments Company, Melville, NY, USA). Figures were assembled using Photoshop software (Adobe, San Jose, California, USA).

Immuno-labelling of Mf-associated monocytes and PMNs was performed on samples of Mf incubated with purified PMNs and monocytes for 120 hours in 96-well round bottomed plates (Corning Glass Works, NY). Samples were centrifuged onto microscope slides at 1000 rpm for 5 min in a Cytospin 3 cytocentrifuge (Shandon Scientific Limited, Astmoor, Runcorn, Cheshire, England), fixed in methanol for 50 second blocked with 5% FBS, 1% BSA in PBS for 30 min. The slides were incubated with a 1:100 dilution of mouse anti-human CD16-Alexa Fluor 488, clone 3GB (Stemcell Technologies, Vancouver, CA) and 1:100 dilution of mouse anti-human CD14 Alexa Fluor 594 clone HCD14 (Biolegend, San Diego, CA) in blocking solution for 2 hours RT in a humidified chamber in the dark. DAPI was added to the primary antibody solution for the last 25 min of incubation. Following rinsing for 2 x 5 min in PBS, the slides were coverslipped, mounted in Mowiol and set overnight prior to viewing. Z-stack images were collected using a Nikon A1R confocal microscope and NIS Elements software (Nikon Instruments Company, Melville, NY, USA). Images were prepared using Adobe Photoshop software (Adobe, San Jose, California, USA).

### Quantification of extracellular DNA release

Assays were set up in Nunc 384-Well optical bottom tissue culture plates (THERMO Scientific, Rochester, NY, USA). There were four components added to each well in 12.5μl volumes, giving a total volume of 50μl. ~18,750 PMNs were added to each well. PMNs were suspended in RPMI-1640 containing 12.5μM SYTOX Orange Nucleic Acid Stain (Life Technologies, Eugene, OR, USA) to give a final concentration of 3.125μM of SYTOX Orange stain and enable the quantification of extracellular DNA [37, 38]. The other three well components varied between treatment groups and included: 5% autologous serum, 5% autologous heat treated serum, ~25 *B*. *malayi* Mf, 10 μM diphenyleneiodonium (DPI; Sigma, St. Louis, MO, USA), 30μg/ml DNase I (Roche, Indianapolis, IN, USA) and 25nM phorbol myristate acetate (PMA; Sigma, St. Louis, MO, USA), which was employed as a positive control. The concentrations stated here represent the final concentrations obtained once all components had been added to the well. To create the negative controls, 12.5μl of RPMI-1640 was substituted for the relevant component. The tissue culture plates were incubated at 37°C and 5% CO_2_ throughout the experiment. Both transmitted light and fluorescence images were captured on a Nikon A1R confocal microscope system equipped with a 60X 1.4NA lens. A single field of view with was taken at a random position within each well. Images were taken every 30 min for 7 hours using automated capture software. The first images were taken 1 hour post-experimental set up to allow the worms and cells to settle to the bottom the wells. Mean SYTOX Orange intensities of the fluorescent images were quantified using the measure region of interest (ROI) feature of the Nikon A1 software. The entire image was highlighted as the ROI. These measurements were used to calculate changes in SYTOX Orange intensities over time (ΔMean SYTOX Orange intensity). Each biological replicate represents the mean of three technical replicates.

### PMN attachment assay

Assays were set up in Nunc 96-Well optical bottom tissue culture plates (THERMO Scientific, Rochester, NY, USA). There were four components added to each well in 50μl volumes, giving a total volume of 200μl. ~100 *B*. *malayi* Mf, ~75,000 PMNs and 50μl of RPMI-1640 were added to each well. The worm to cell ratio selected (1:750) was sufficiently high so that the number of available cells did not limit attachment [14]. The final component added to each well varied between treatment groups and included: 5% autologous serum, 5% autologous heat treated serum, 10μM DPI and 30μg/ml DNase I. To create the respective controls, 50μl of RPMI-1640 was substituted for the relevant component. The tissue culture plates were incubated at 37°C and 5% CO_2_ throughout the experiment. Videos of each well were taken on an inverted microscope (40X magnification) at 2.5, 5, 16 and 24 hours post-experimental set up. Videos were blinded and all Mf were scored for attachment. Mf that had at least 1 PMN adhered to their surface or indirectly fastened by extracellular DNA were scored as attached. Each biological replicate represents the mean of two or three technical replicates. PMN attachment to Mf was confirmed by cytocentrifugation and Wright stain as described above.

### Mf survival assay

Assays were set up in Nunc 96-Well optical bottom tissue culture plates. There were four components added to each well in 50μl volumes, giving a total volume of 200μl. ~100 *B*. *malayi* Mf were added to each well. The other three components varied between treatment groups and included: 25% autologous serum or 25% autologous heat treated serum, ~150,000 PMNs, ~150,000 PBMCs, ~150,000 monocytes, ~150,000 lymphocytes and 30μg/ml DNase I. To create the respective controls, 50μl of RPMI-1640 was substituted for the relevant component. The tissue culture plates were incubated at 37°C and 5% CO_2_ throughout the experiment. Videos of each well were taken on an inverted microscope (40X magnification) at 1, 24, 48 and 120 hours post-experimental set up. Videos were blinded and the numbers of moving Mf present within each well were counted. Mf that were not moving were considered dead. The number of surviving Mf was normalized relative to the number of moving Mf scored 1 hour post-set up (= 100%), and expressed as a relative percentage. Each biological replicate represents the mean of two or three technical replicates.

### Compstatin- and antibody-treated serum

For the compstatin inhibition studies, autologous serum was pretreated with either 100μM compstatin (Tocris Bioscience, Avonmouth, Bristol, U.K.) or 100μM compstatin control peptide (Tocris Bioscience, Avonmouth, Bristol, U.K.) for 30 min at 37°C. The compstatin treated serum and antibody treated leukocytes were added directly to the PMN attachment and Mf survival assays described above. Inhibition of Cd11b activity was implemented by incubating 2g of the monoclonal antibody clone M1/70 purified specifically for use with live cells (BioLegend, San Digeo, CA, cat #101248) with PMNs for 2 hours at room temperature prior to adding *B*.*malayi* Mf and incubating for 5 days at 37°C prior to assessing survival as described above. Block of ICAM-1 function was carried out as for Cd11b using 2g of anti-ICAM1 antibody [MEM-11] (Abcam, Cambridge, MA).

### Data analysis

Normality of the data was assessed based on examination of histograms and normal Q-Q plots of the residuals. Constant variance of the data was assessed by plotting residuals against predicted values. Data were analyzed using linear mixed effects modeling with independent experiment modeled as a random effect and treatment group and time (when applicable) modeled as fixed nominal effects. Two-way interactions were also included in the model when applicable. Model fit was assessed using Akaike information criterion values. When indicated, adjustments for multiple relevant comparisons were done using the method of Bonferroni. For all analyses, adjusted P<0.05 was considered significant.

## Supporting Information

S1 Video Video capture of Mf tethered within PMN extruded extracellular DNA. Mf and PMN were co-incubated in the presence of SYTOX Orange and videos recorded 16 hours post-incubation. SYTOX Orange fluorescence in white light appears pink and can be seen in concentrated areas associated with several Mf.
(AVI)

S2 Video Transmitted light and fluorescence images of co-incubated Mf and PMN. Mf and PMN were co-incubated in the presence of SYTOX Orange and confocal images acquired every 30 min for 7 hours. The right hand panel is SYTOX only, middle transmitted light only and left hand panel is the combination of fluorescence and transmitted light channels showing an increase in association of extracellular DNA with Mf over time that is characteristic of a spread NET morphology.
(AVI)

S1 Fig The effect of serum and heat-treated serum on extracellular DNA release from PMN in the presence of PMA. (TIF)
